# FDG and FMISO PET-guided dose escalation with intensity-modulated radiotherapy in lung cancer

**DOI:** 10.1186/s13014-018-1147-2

**Published:** 2018-10-23

**Authors:** Sébastien Thureau, Bernard Dubray, Romain Modzelewski, Pierre Bohn, Sébastien Hapdey, Sabine Vincent, Elodie Anger, David Gensanne, Nicolas Pirault, Gouel Pierrick, Pierre Vera

**Affiliations:** 1 Department of Radiation Oncology and Medical Physics, Centre Henri Becquerel, QuantIF – LITIS [EA 4108], Université de Normandie, CS 11516, rue d’Amiens, 76038 Rouen Cedex 1, France; 2 Department of Nuclear Medicine, Henri Becquerel Cancer Center and Rouen University Hospital, & QuantIF – LITIS [EA (Equipe d’Accueil) 4108 – FR CNRS 3638], Faculty of Medicine, University of Rouen, Rouen, France

**Keywords:** Positron emission tomography, ^18^fluoro-deoxy-D-glucose, ^18^fluoro-misonidasole, Lung cancer, Hypoxia, Radiotherapy

## Abstract

**Background:**

Concomitant chemo-radiotherapy is the reference treatment for non-resectable locally-advanced Non-Small Cell Lung Cancer (NSCLC). Increasing radiotherapy total dose in the whole tumour volume has been shown to be deleterious. Functional imaging with positron emission tomography (PET/CT) offers the potential to identify smaller and biologically meaningful target volumes that could be irradiated with larger doses without compromising Organs At Risk (OAR) tolerance. This study investigated four scenarios, based on ^18^FDG and ^18^F-miso PET/CT, to delineate the target volumes and derive radiotherapy plans delivering up to 74Gy.

**Method:**

Twenty-one NSCLC patients, selected from a prospective phase II trial, had ^18^FDG- and ^18^F-miso PET/CT before the start of radiotherapy and ^18^FDG PET/CT during the radiotherapy (42Gy). The plans were based planned on a standard plan delivering 66 Gy (plan 1) and on three different boost strategies to deliver 74Gy total dose in pre-treatment ^18^FDG hotspot (70% of SUV_max_) (plan 2), pre-treatment ^18^F-miso target (SUV_max_ > 1.4) (plan 3) and per-treatment ^18^FDG residual (40% of SUV_max_). (plan 4).

**Results:**

The mean target volumes were 4.8 cc (± 1.1) for ^18^FDG hotspot, 38.9 cc (± 14.5) for ^18^F-miso and 36.0 cc (± 10.1) for per-treatment ^18^FDG. In standard plan (66 Gy), the mean dose covering 95% of the PTV (D95%) were 66.5 (± 0.33), 66.1 (± 0.32) and 66.1 (± 0.32) Gy for ^18^FDG hotspot, ^18^F-miso and per-treatment ^18^FDG. In scenario 2, the mean D95% was 72.5 (± 0.25) Gy in ^18^FDG hotspot versus 67.9 (± 0.49) and 67.9 Gy (± 0.52) in ^18^F-miso and per-treatment ^18^FDG, respectively. In scenario 3, the mean D95% was 72.2 (± 0.27) Gy to ^18^F-miso versus 70.4 (± 0.74) and 69.5Gy (± 0.74) for ^18^FDG hotspot and per-treatment ^18^FDG, respectively. In scenario 4, the mean D95% was 73.1 (± 0.3) Gy to ^18^FDG per-treatment versus 71.9 (± 0.61) and 69.8 (± 0.61) Gy for ^18^FDG hotspot and ^18^F-miso, respectively. The dose/volume constraints to OARs were matched in all scenarios.

**Conclusion:**

Escalated doses can be selectively planned in NSCLC target volumes delineated on ^18^FDG and ^18^F-miso PET/CT functional images. The most relevant strategy should be investigated in clinical trials.

**Trial registration:**

(RTEP5, NCT01576796, registered 15 june 2012)

## Background

Non-small cell lung cancer (NSCLC) is a deadly disease. The majority of non-metastatic NSCLC cannot undergo surgical resection with curative-intent, either due to the patient’s medical condition or to cancer local extension. Concomitant Radiotherapy - Chemotherapy (RTCT) is the standard curative-intent treatment for non-operable patients/non resectable cancers [1]. Large efforts in radiotherapy techniques are made to improve tumour control and survival [1, 2]. A total dose > 60 Gy to the entire tumour volume defined on CT or PET was deleterious in the RTOG 0617 randomized trial [3]. Many relapses occur within the radiotherapy target volume, suggesting insufficient total dose [4, 5]. Reducing the target volume to high recurrence risk areas is assumed to allow isotoxic dose escalation. Experimental and clinical data have shown that tumour subvolumes defined by high ^18^F-FDG (metabolic hotspot) or ^18^F-misonidazole (^18^F-miso) uptake are associated to recurrence and cancer death [6].

In a phase II study (NCT01576796, RTEP5 study), we used ^18^F-miso to identify and delineate hypoxic areas within the ^18^FDG-defined Gross Target Volume (GTV) [7]. The total radiotherapy dose was safely increased in 24 out of 34 patients with ^18^F-miso uptake. Doses up to 86 Gy could not reverse the poor prognosis features of ^18^F-miso positive tumours. In this study, hypoxic tumours with boost had the same local control despite twice as large volumes. The patients’ data were used to test in silico three strategies for selective increase in total dose, based on functional imaging. For the strategies with boost, we tested at a dose of 74 Gy to be comparable to the study of Bradley et al. [3] and our current phase II/III (RTEP7; NCT02473133). In 21 patients, a standard plan (66 Gy to whole Planning Target Volume PTV plan 1) to 74 Gy was compared to the pre-RT ^18^FDG metabolic hotspot (70% of SUVmax) (plan 2), 2/ the pre-RT ^18^F-MISO-affine (SUV > 1.4) volume (plan 3) or the per-treatment ^18^FDG uptake (40% of SUVmax) (plan 4).

## Methods

### Study design and patients

**Fig. 1:**
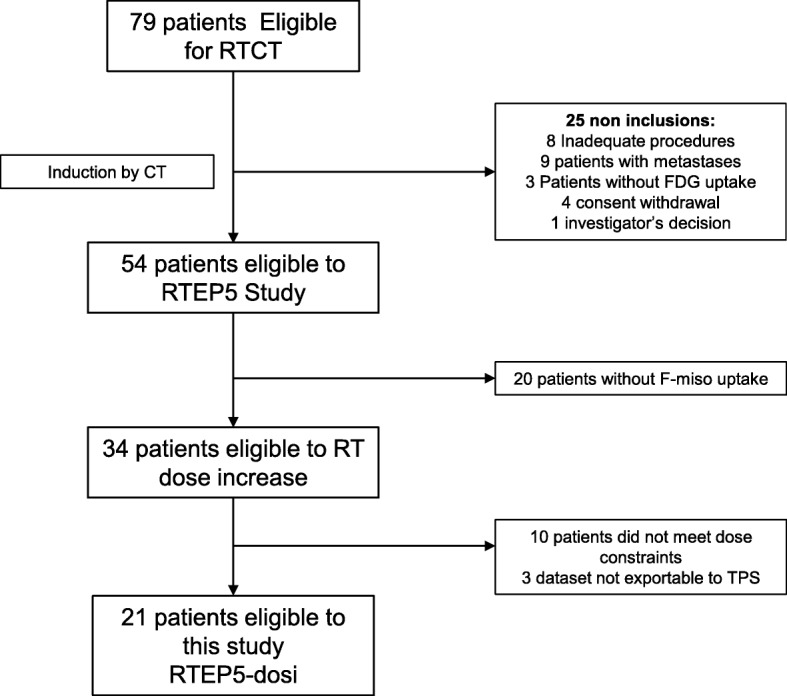
Study design. RTCT: radio-chemotherapy; TPS: Treatment Planning System

The details of the study can be found elsewhere [7]. Fifty-four patients with NSCLC, eligible for curative-intent RTCT, and with significant FDG uptake on pre-RT PET/CT were prospectively selected. The 21 patients with significant per-RT F-miso uptake and meeting the dose/volume constraints for the organs at risk (OAR) form the basis of the present study (Fig. 1). All the patients had signed a written consent to participate to RTEP5.

### PET/CT imaging

Two ^18^FDG PET/CT (FDG_1_ and FDG_2_) and two ^18^F-miso PET/CT (F-miso_1_ and F-miso_1_) acquisitions were performed before and during RT using the same machine and under the identical operational conditions, a centrally supervised quality control securing homogeneity in the image quality in all centres [7]. FDG_1_ was acquired in treatment position (arms over the head, free breathing), at least 15 days after the last administration of chemotherapy. F-miso_1_ was scheduled 48 h after the FDG1. The F-miso PET/CT acquisitions were reviewed by 3 independent experts (out of the 9 experts) who decided upon the presence or absence of uptake [8]. The CT scan images were used to register all PET/CT acquisitions, delineate target volumes and plan radiotherapy. Respiratory-gated 4D acquisitions were not performed. No further chemotherapy was allowed between FDG_1_/F-miso_1_ and the start of radiotherapy. The F-miso_2_ was not associated with a planification due to the very low contrast in per-treatment [7].

### Target volume definition

For each patient, the CTs of FDG_1_, FDG_2_ and F-miso_1_ were co-registered to the planning CT scanner (Oncoplanet, DosiSoft, France, v 1.4), focusing on the tumour. The GTV for FDG_1_ was defined as the sum of the voxels with uptake > 40% of the SUVmax inside primary tumour or nodes and corrected from CT data. The ^18^FDG hotspot was defined as all voxels with uptake > 70% of the SUVmax inside the primary tumour or nodes (BTVm1). The hypoxic volume (BTVh) was defined as the sum of voxels with SUV ≥1.4 on F-miso_1_ [7, 8]. The residual volume on FDG PET/CT2 (BTVm2) was defined as the sum of the voxels with uptake > 40% of the SUVmax inside the primary tumour or nodes. The co-registered volumes were then transferred to an Eclipse planning platform (V13.6, Varian Inc.). The CTV66 were obtained either by isotropic expansion around the primary tumour (6 mm for squamous cell carcinoma, 8 mm for adenocarcinoma), then manually edited to exclude the bones, the large vessels and heart, the muscle and the trachea, or by anatomical delineation of the involved nodal stations [9, 10]. The margins around the BTVs or CTVs to delineate the Planning Target Volume (PTV) were 10 to 15 mm. All delineation were performed by an experienced radiation oncologist (ST).

### Planning scenarios

Four IMRT by step and shoot scenarios were applied in each patient. All the dose calculations were corrected for heterogeneity (V13.6, AAA v10.0.28 Varian Inc.) and for the optimization (DVO v10.0.28, Varian Inc). The total dose was prescribed at ICRU point, the dose delivered in the PTV having to be within 95% and 107% of the prescribed dose. Absolute priority constraints were a maximum dose to the spinal cord < 45 Gy and no more than 30% of the total lung volume (excluding the PTV) receiving ≥20 Gy (V20Gy). The secondary dose/volume constraints were no more than 30% of the oesophagus or the heart receiving ≥50 or ≥ 35 Gy, respectively.

The first scenario (standard or reference plan; plan 1) was to deliver 66 Gy in the PTV based on FDG1. The experimental scenarios had to deliver 66 Gy in the FDG1 PTV (PTV66) and an additional dose up to 74 Gy in three different smaller target volumes. The boost target volumes were the metabolic hotspot on pre-treatment FDG PET/CT (BTVm1, scenario 2), the hypoxic volume on pre-treatment F-miso PET/CT (BTVh, scenario 3), and the residual uptake on the FDG PET/CT at 42 Gy (BTVm2, scenario 4). The treatment was planned with a simultaneous boost from the start of the radiotherapy for plan 2 and 3 and from 50 Gy for the plan 4.

### Statistical analyses

Descriptive statistics (n, mean, SE minimum and maximum) were calculated for the quantitative variables. Frequency and percentages with 95% confidence intervals (CI) were computed for the qualitative variables. Levene’s test was used to assess variances equality when comparing the quantitative variables means between two or more groups (ANOVA). All statistical calculations were performed with MedCalc Software (version16.2.0, Ostend, Belgium).

## Results

### Population

The present study was based on 21 patients (4 women and 20 men, mean age (±SE) = 59 ± 8 years). There were 7 adenocarcinomas, 12 squamous cell carcinomas and 2 undifferentiated carcinomas. The stages distribution was 1 IIB, 12 IIIA and 8 IIIB (Table 1).

**Table 1:** Baseline characteristics of 21 included patients

Patient	Gender	Age	TNM	Stage	Pathology	Volume (cm3)				
						CTV 66Gy	BTV FDG 40%	BTV FDG 70%	BTV FMISO	BTV FDG perRT
1	F	51	T3N3M0	IIIB	Adenocarcinoma	157.2	21.1	3.0	1.9	18.7
2	M	64	T4N0M0	IIIA	Squamous cell carcinoma	512.6	186.5	6.9	75.3	162.0
3	F	59	T1N2M0	IIIA	Adenocarcinoma	68.5	9.9	7.6	2.4	2.3
4	M	51	T1N3M0	IIIB	Adenocarcinoma	45.8	15.1	2.4	0.1	5.1
5	M	59	T3N3M0	IIIB	Adenocarcinoma	273.2	87.2	6.4	8.3	78.7
6	F	60	T3N2M0	IIIA	Adenocarcinoma	648.7	189.5	6.1	143.1	132.1
7	M	76	T2N0M0	IIA	Squamous cell carcinoma	56.8	13.8	5.0	2.3	2.8
8	F	62	T2N2M0	IIIA	Squamous cell carcinoma	116.3	6.3	0.2	4.4	1.1
9	M	63	T4N1M0	IIIA	Squamous cell carcinoma	236.1	19.2	0.2	5.7	8.9
10	M	65	T4N2M0	IIIB	Squamous cell carcinoma	157.4	14.9	0.3	26.1	9.1
11	M	72	T3N2M0	IIIA	Squamous cell carcinoma	289.4	101.1	19.1	272.5	75.8
12	M	59	T4N3M0	IIIB	Squamous cell carcinoma	290	37.2	7.6	36.8	15.4
13	M	55	T4N2M0	IIIB	Squamous cell carcinoma	970.9	353.2	6.7	100.8	87.6
14	M	58	T4N0M0	IIIB	Squamous cell carcinoma	283.7	72.6	15.1	81.1	34.0
15	M	45	T3N2M0	IIIA	Squamous cell carcinoma	80.5	30.5	0.4	16.1	9.2
16	M	54	T2N2M0	IIIA	Adenocarcinoma	64.4	9.4	0.6	2.4	5.1
17	M	61	T4N2M0	IIIB	Squamous cell carcinoma	241.1	22.6	0.9	24.2	64.4
18	M	58	T3N0M0	IIB	Unknow	90.4	8.8	2.5	7.9	9.1
19	M	63	T2N2M0	IIIA	Adenocarcinoma	328.8	9.5	0.5	1.8	10.2
20	M	70	T3N2M0	IIIA	Squamous cell carcinoma	135	24.3	1.6	0.2	4.8
21	M	42	T0N2M0	IIIA	Unknow	82.1	29.4	8.0	2.8	19.7
mean		**59**				**244.2**	**60.1**	**4.8**	**38.9**	**36**
SE		*8*				*49.6*	*18.8*	*1.1*	*14.5*	*10.1*

### Target volumes and dose distribution

The mean CTV_66Gy_ was 244 ± 50 cc, larger than all BTVs. The ^18^FDG hotspot (BTV FDG 70%) smaller than the BTV FDG per-RT (4.8 ± 1.1 versus 36 ± 10 cc, *p* = 0.03) and the BTV hypoxic (39 ± 15 cc, *p* = 0.13). Similarly, the mean PTV_66Gy_ was 473 ± 69 cc versus 35 ± 5.8 cc for PTV FDG 70%, 111 ± 24 cc for PTV FDG per-RT, and 105 ± 26 cc for PET F-miso (Table 1).

**Fig. 2:**
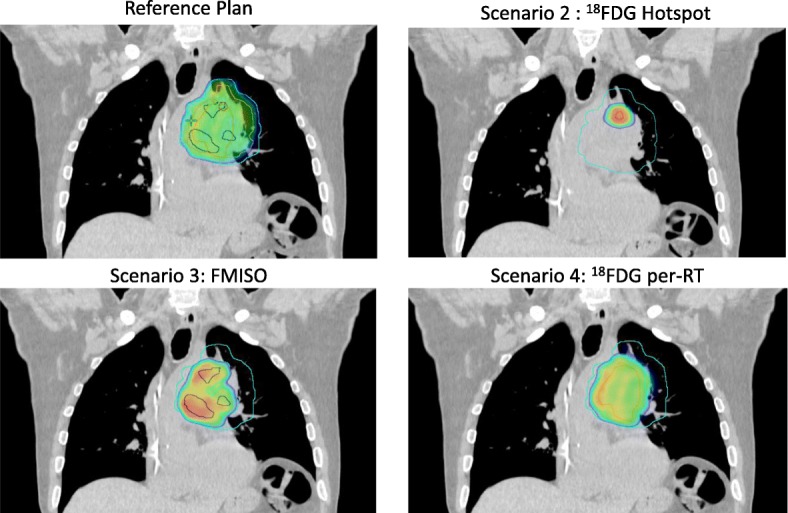
Example of dosimetry. Representation of dose (95% to maximum dosi) fr reference plan (66Gy) and three scenario of planification based on PET. PTV are blue, hotspot (BTVm1) is red, FDG pre-RT (BTVm2) is orange and FMISO (BTVh) is dark blue. For each plan the PTV 66 is represented

In scenarios 2, 3 and 4, the mean doses to the specific target volume was higher than the doses given to the other biological volumes or PTV 66 Gy. In scenario 2 (boost to ^18^FDG hotspot), the mean dose to 95% of the PTV (D95%) was 72.5 ((± 0.25) Gy versus 67.9 (± 0.49) (*p* < 0.0001) in F-miso PTV and 67.9 Gy (± 0.52) (*p* < 0.0001) in per-treatment ^18^FDG PTV. In scenario 3 (boost to ^18^F-miso), the mean D95% was 72.2 (± 0.27) Gy versus 70.4 (± 0.33) (*p* = 0.74) in ^18^FDG hotspot and 69.5 (± 0.74) Gy (*p* = 0.009) in per-treatment ^18^FDG PTV. In scenario 4 (boost to per-treatment ^18^FDG), the mean D95% was 73.1 (± 0.3) Gy versus 71.9 (± 0.61) (*p* = 0.2) in ^18^FDG hotspot and 69.8 (± 0.61) Gy (*p* = 0.0001) to FMISO PTV (Fig. 2). The results are presented for CTV (Table 2), PTV (Table 3) and Organs at Risk (Table 4).

**Table 2:** Comparison of dose to PTV for 4 planning treatment: PTV66, PTV FDG 70%, PTV F-miso, PTV FDG 42 Gy

Scenario	Boost target volume	Mean doses (SE) to target volumes				
		PTV66	PTV FDG 70%	PTV FMISO	PTV FDG 42Gy	*p* value
Reference plan (66Gy)		**62.3** (± 0.38)	66.5 (± 0.33)	66.1 (± 0.32)	66.1 (± 0.32)	*NA*
Scenario 2	FDG Hotspot	62.7 (± 0.4)	**72.5** (± 0.25)	67.9 (± 0.49)	67.9 (± 0.52)	*0.001*
Scenario 3	Fmiso	62.6 (± 0.42)	70.4* (± 0.74)	72.2* (± 0.27**)**	69.5 (± 0.74)	*0.001*
Scenario 4	FDG per treatment	63.7 (± 0.36)	71.9* (± 0.61)	69.8 (± 0.61)	73.1* (± 0.3)	*0.001*

Data in boldface is the reference dose by plan

*no significant difference

**Table 3:** Comparison of dose to CTV or BTV for 4 planning treatment: CTV66, BTV FDG 70%, BTV F-miso, BTV FDG 42 Gy

Scenario	Boost target volume	Mean (± SE) doses to target volumes (Gy)				
		CTV66	BTV FDG 70%	BTV FMISO	BTV FDG 42Gy	*p value*
Reference plan (66 Gy)		**65.1** (± 0.35)	67.1 (± 0.32)	66.9 (± 0.38)	66.8 (± 0.39)	*NA*
Scenario 2	^18^FDG Hotspot	65.9 (± 0.47)	**74.3** (± 0.32)	70.4 (± 0.86)	70.4 (± 0.88)	*0.001*
Scenario 3	^18^F-miso	65.9 (± 0.53)	72.6 (± 0.86)	**74.6** (± 0.33)	71.7 (± 0.9)	*0.001*
Scenario 4	^18^FDG per RT	66.8 (± 0.42)	73.5 (± 0.68)	72.5 (± 0.7)	**74.6** (± 0.31)	*0.001*

Data in boldface is the reference dose by plan

**Table 4:** Comparison of dose to organs at risk for 4 planning treatment: PTV66, PTV FDG 70%, PTV F-miso, PTV FDG 42 Gy

Scenario	Boost target volume	Dose-volume constraint to organ at risk			
		Mean lung dose (Gy)	D2% spinal cord (Gy)	V35 heart (%)	V50 oesophagus (%)
Reference plan		11.5 (±0.5)	32.7 (±2.1)	5** (±1.7)	20.5 (±2.7)
Scenario 2	FDG Hotspot	11.7 (±0.5)	32 (±2.1)	5.3 (±1.8)	20.4 (±2.6)
Scenario 3	Fmiso	11.8 (±0.5)	31.6 (±2.1)	5.2 (±1.9)	21 (±2.6)
Scenario 4	FDG per treatment	13.4 (±1.5)	32.2 (±2.3)	5.4** (±1.8)	20.8 (±2.7)
*p* value		0.2	0.7	0.03	0.4

**significant difference

The dose/volume constraints to the organs at risk were matched without significant differences between scenarios except to the heart between the plan to 66 Gy and FDG per-treatment plan with V35 at respectively 5% (± 1.7) and 5.4% (± 1.8) (*p* = 0.05) (Table 4).

## Discussion

The present planning study confirms that selecting various sub-volumes to increase radiotherapy total dose results in different dose distributions. The data of 21 patients were retrieved from a prospective phase II study investigating the clinical feasibility of boosting the radiotherapy dose in tumour hypoxic areas delineated on ^18^F-miso PET/CT. Pre- and per-radiotherapy ^18^FDG allowed us to compare three different biologically-oriented strategies.

RTCT is the reference treatment for locally advanced non operable NSCLC. The high incidence of relapse within the target volume suggest insufficient total doses of radiotherapy to achieve local control. The RTOG 0617 randomized trial [3] demonstrated that an indiscriminate dose increase in all patients and to the entire ^18^FDG PET/CT volume was deleterious. The combination of functional information (metabolism or hypoxia) and improved radiotherapy delivery (IMRT) opens the way to selectively increase total dose in biologically-relevant parts of the tumour. In a previous study, we showed that areas of high ^18^FDG uptake (SUV > 70% SUVmax) on pre-treatment PET/CT scans were associated to tumour areas at greater risk of relapse [11]. Similar results have been reported in NSCLC [12] and in oesophageal cancer patients [13]. A European randomized phase II is currently investigating an integrated boost up to 72 Gy in the > 50% SUV_max_ volume delineated on pre-treatment ^18^FDG PET/CT (PET Boost; NCT01024829). We are presently conducting a phase II study (RTEP7; NCT02473133) where the radiotherapy dose is escalated up to 74 Gy in the metabolic residual as assessed on FDG-PET/CT performed at 42 Gy. Targeting the hypoxic volume as identified by ^18^F-miso PET/CT before or during radiotherapy was investigated in RTEP5 [7] and RTOG-1106 (NCT01507428) trials, respectively, as well as in head and neck cancer patients [14, 15].

The three scenarios investigated here yielded different target volumes for radiotherapy dose escalation. Our data contradict the idea that ^18^FDG uptake is associated to the presence of hypoxia via the upregulation of glucose transporter 1 by hypoxia-inducible factor 1 [16, 17]. ^18^FDG and ^18^F-miso provide different and possibly complementary information. Given the impact on dose distribution as observed here, the selection of the most relevant strategy will rely on clinical trials. In our RTEP5 phase II [7], the patients with significant 18F-miso uptake had worse disease-free and overall survival probabilities. This observation questions the assumption that hypoxia-related radio-resistance could be overcome by moderate additional doses targeted to the hypoxic volume. On the other hand, most of the dose escalation trials (including our RTEP5 phase II) achieved higher doses by adding several 2-Gy fractions to the reference irradiation schedule. The protraction of radiotherapy (up to 7.5 weeks in RTOG 0617) is known to favour tumour cell proliferation and to reduce the probability of tumour control. In our ongoing RTEP7 phase II, the boost is given by fractions of 3 Gy to avoid longer treatment times. In the present scenarios, IMRT allows to delivered simultaneous integrated boost (2 Gy per fraction in PTV66 and 2.24 Gy per fraction in the boost defined on pre-treatment PET/CT. Note that scenario 4 requires a 2-step planning (up to 50 Gy, then up to 74 Gy with 8 fractions of 3 Gy. Other approaches could be proton therapy [18] or stereotaxic radiotherapy [19].

Non respiratory-gated images for planning CT and PET/CT could have hampered the delineation precision and strategy for reducing the PTV margins. When the RTEP5 study was initiated, 4D acquisitions (and IMRT) were not routinely performed in the participating centres. The decision was made to require ungated acquisitions (and 3D conformal radiotherapy) in order to secure our recruitment objectives and the exportability of our results. The majority of our patients had large stage III tumours involving the mediastinum, limiting breathing movements. In addition, our delineation criteria on 18F-miso PET/CT was validated in free-breathing patients [8]. Precise radiotherapy (4D, IMRT) is indispensable if you want to target tumoral sub-volumes.

Patient selection is another issue. Positive ^18^F-miso uptake was associated to worse prognosis in our previous RTEP5 study [7]. Ten patients, out of 34 with ^18^F-miso uptake, were not eligible to the present planning study, mostly because of too large target volumes precluding dose escalation without compromising OAR tolerance. Beyond feasibility studies, randomised trials are warranted to demonstrate the value of radiotherapy assisted by functional imaging.

## Conclusion

Pre−/per-treatment ^18^FDG and pre-treatment ^18^F-miso PET/CT yield different candidate target volumes for selective increase in radiotherapy dose in patients with NSCLC. Our in-silico study shows that IMRT provides radiotherapy plans matching the pre-defined dose/volume objectives and constraints. Clinical trials are required to select the relevant strategies to improve outcome after concomitant chemo-radiotherapy.

## Abbreviations

BTV: Biologic Target Volume
GTV: Gross Target Volume
IMRT: Intensity modulated Radiation Therapy
NSCLC: Non-small cell lung cancer
OAR: Organs at risk
PTV: Planning Target Volume
RTCT: Radiotherapy - Chemotherapy
